# The chromosomal genome sequence of the sponge *Phakellia ventilabrum *(Linnaeus, 1767) and its associated microbial metagenome sequences

**DOI:** 10.12688/wellcomeopenres.25431.1

**Published:** 2026-01-06

**Authors:** Sergi Taboada, Ana Riesgo, Kathrin Busch, Dirk Erpenbeck, Ute Hentschel, Carles Galià, Graeme Oatley, Elizabeth Sinclair, Eerik Aunin, Noah Gettle, Camilla Santos, Michael Paulini, Haoyu Niu, Victoria McKenna, Rebecca O’Brien, Wellcome Sanger Institute Tree of Life Management, Samples and Laboratory Team, Wellcome Sanger Institute Scientific Operations: Sequencing Operations, Wellcome Sanger Institute Tree of Life Core Informatics Team, EBI Aquatic Symbiosis Genomics Data Portal Team, Aquatic Symbiosis Genomics Project Leadership

**Affiliations:** 1 Department of Biodiversity and Evolutionary Biology, MNCN-National Museum of Natural Sciences, Madrid, Spain; 2 GEOMAR Helmholtz Centre for Ocean Research Kiel, Kiel, Germany; 3 University of New South Wales, Sydney, Australia; 4 Department of Earth and Environmental Sciences, Palaeontology & Geobiology, LMU München, Munich, Germany; 5 Tree of Life, Wellcome Sanger Institute, Hinxton, England, UK

**Keywords:** Phakellia ventilabrum, genome sequence, chromosomal, Bubarida, microbial metagenome

## Abstract

We present a genome assembly from a specimen of *Phakellia ventilabrum* (Porifera; Demospongiae; Bubarida; Bubaridae). The genome sequence has a total length of 211.92 megabases. Most of the assembly (99.97%) is scaffolded into 25 chromosomal pseudomolecules. The mitochondrial genome has also been assembled and is 24.36 kilobases in length. Gene annotation of this assembly by Ensembl identified 21 622 protein-coding genes. Thirty-three binned genomes were generated from the metagenome assembly, of which eight were classified as high-quality metagenome assembled genomes (MAGs) and of which four of the MAGs are fully circular. The MAGs were taxonomically assigned to Pseudomonadota (i.e. Candidatus Poriferihabitaceae), Nitrospirota, Nitrospinota, and the archaeal Nitrosopumilus clade.

## Species taxonomy

Eukaryota; Opisthokonta; Metazoa; Porifera; Demospongiae; Heteroscleromorpha; Bubarida; Bubaridae; *Phakellia*; *Phakellia ventilabrum* (Linnaeus, 1767) (NCBI:txid942649)

## Background

*Phakellia ventilabrum* (Linnaeus, 1767), is a deep-water demosponge, common on rock-sand habitats across the North Atlantic Ocean, although it has also been reported in the Western Mediterranean Sea ( de Voogd *et al.*, 2024; Maldonado *et al.*, 2017; Sánchez *et al.*, 2009). This fan-shaped axinellid sponge has a wide bathymetric distribution from shallow waters of 10 m to depths reaching almost 1,900 m ( Prado *et al.*, 2020). *Phakellia ventilabrum* can form dense aggregations and is considered as a Vulnerable Marine Ecosystem (VME) indicator species ( Maldonado *et al.*, 2017).

*Phakellia ventilabrum* is an oviparous and gonochoristic (i.e. separate sexes) species, reproducing in May and September, with potentially lecithotrophic larva or direct development, given the relatively high amount of nutrients (mainly lipids) accumulated in the egg during vitellogenesis ( Koutsouveli *et al.*, 2022). Its dispersal ability and the main oceanographic currents in the North Atlantic Ocean drive the genetic connectivity and genetic diversity patterns of this species. Two distinct genetic populations have been identified, one comprising individuals from the Cantabrian Sea to Roscoff, and another comprising sponges from the south of the British Islands up to the north of Norway ( Taboada *et al.*, 2023). The genetic break detected in this genome-wide analysis is explained by oceanographic models and indicated a high degree of isolation. The low genetic diversity for the Cantabrian *P. ventilabrum* might result in compromised resilience for this population considering the current climate change scenario predictions for the southern areas ( Taboada *et al.*, 2023).

*Phakellia ventilabrum* is an optimal natural sampler for environmental DNA (eDNA). Collections along the North Atlantic have identified unprecedented levels of metazoan diversity within this species, with more than 400 species detected by eDNA methods ( Gallego *et al.*, 2024; Neave *et al.*, 2023). Because *P. ventilabrum* is a Low Microbial Abundance (LMA) species, its presumed high filtration rates may be favourable for accumulating a higher amount of eDNA ( Weisz *et al.*, 2008). The microbiome of *P. ventilabrum* is dominated by Pseudomonadota and Nitrospirota and it displays sponge species-specificity in comparison to closely-related species from the same collection sites ( Busch *et al.*, 2022; Taboada *et al.*, 2022).

The availability of the chromosome-level genome of *P. ventilabrum* will help to further explore population connectivity and adaptation patterns of this key-stone species, which will have direct implications for conservation purposes. The availability of another chromosome-level genome for *P. ventilabrum* ( Mc Cartney *et al*., 2024) now provides a wealth of data for comparative genomic analyses to address evolutionary and ecological questions with unprecedented detail. In addition, this genome will be particularly useful for investigating the evolution of biological traits in Demospongiae as well as for clarifying the phylogenetic relationships within the family Bubaridae. This last case is especially relevant for the polyphyletic genus *Phakellia* ( Morrow *et al.*, 2012; Redmond *et al.*, 2013; Taboada *et al.*, 2022) and with *P. ventilabrum* representing the type species. Finally, the microbial genomic data provided here will enable future studies on the microbial composition and functionality of this species across its geographic and phylogenetic distribution, ( Hentschel *et al.*, 2012; Díez-Vives & Riesgo, 2024; Thomas *et al.*, 2016).

## Genome sequence report

### Sequencing data

**Figure 1.**
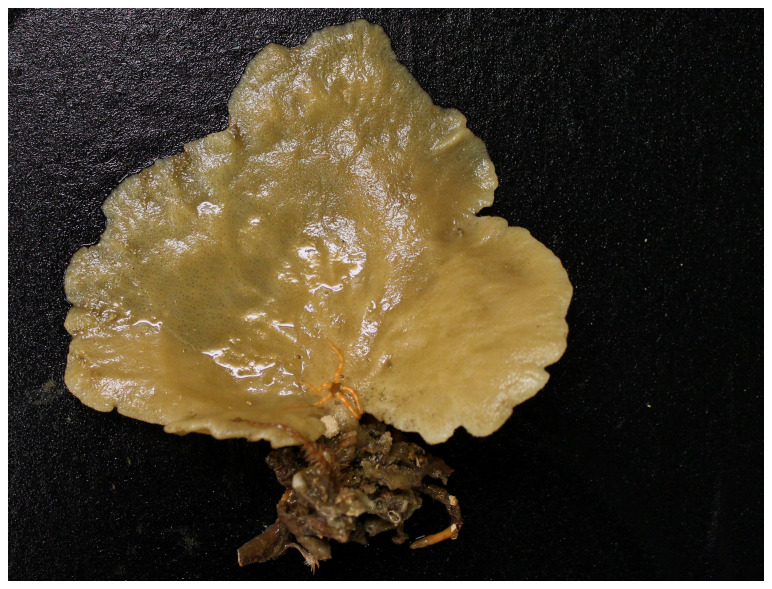
*Phakelia ventilabrum* specimen used for genome sequencing. The image depicts the specimen immediately after collection onboard ship and prior to deep-freezing. The size of the sponge body is about 20–25 cm in diameter. Image taken by Kathrin Busch.

The genome of a specimen of *Phakellia ventilabrum* ( Figure 1) was sequenced using Pacific Biosciences single-molecule HiFi long reads, generating 26.14 Gb from 2.84 million reads. Based on the estimated genome size, the sequencing data provided approximately 93 coverage of the genome. Chromosome conformation Hi-C data produced 116.05 Gb from 768.51 million reads. RNA sequencing data were also generated and are available in public sequence repositories. Table 1 summarises the specimen and sequencing information.

**Table 1.** Specimen and sequencing data for *Phakellia ventilabrum*.

Project information			
**Study title**	Phakellia ventilabrum		
**Umbrella BioProject**	PRJEB78906		
**Species**	*Phakellia ventilabrum*		
**BioSpecimen**	SAMEA115470870		
**NCBI taxonomy ID**	942649		
Specimen information			
**Technology**	**ToLID**	**BioSample accession**	**Organism part**
**PacBio long read sequencing**	odPhaVent3	SAMEA115470889	Somatic tissue
**Hi-C sequencing**	odPhaVent3	SAMEA115470888	Somatic tissue
**RNA sequencing**	odPhaVent3	SAMEA115470887	Somatic tissue
Sequencing information			
**Platform**	**Run accession**	**Read count**	**Base count (Gb)**
**Hi-C Illumina NovaSeq X**	ERR13494022	7.69e+08	116.05
**PacBio Revio**	ERR13510315	2.84e+06	26.14
**RNA Illumina NovaSeq X**	ERR14711437	7.37e+07	11.13

### Assembly statistics

The primary haplotype was assembled, and contigs corresponding to an alternate haplotype were also deposited in INSDC databases. The assembly was improved by manual curation, which corrected 26 misjoins or missing joins and removed 6 haplotypic duplications. These interventions reduced the total assembly length by 4.22%, decreased the scaffold count by 83.33%, also decreasing the scaffold N50 by 0.58%. The final assembly has a total length of 211.92 Mb in 26 scaffolds, with 59 gaps, and a scaffold N50 of 8.44 Mb ( Table 2).

**Table 2.** Genome assembly data for *Phakellia ventilabrum*.

Genome assembly	
Assembly name	odPhaVent3.1
Assembly accession	GCA_964276705.1
*Alternate haplotype accession*	*GCA_964276595.1*
Assembly level for primary assembly	chromosome
Span (Mb)	211.92
Number of contigs	85
Number of scaffolds	26
Longest scaffold (Mb)	22.06
Assembly metric	Measure
Contig N50 length	5.01 Mb
Scaffold N50 length	8.44 Mb
Consensus quality (QV)	Primary: 63.4; alternate: 61.4; combined 62.3
BUSCO *	C:79.7%[S:78.7%,D:0.9%],F:8.4%,M:11.9%,n:954
Percentage of assembly assigned to chromosomes	99.98%
Organelles	Mitochondrial genome: 24.36 kb

* BUSCO scores based on the metazoa_odb10 BUSCO set using version 5.5.0. C = complete [S = single copy, D = duplicated], F = fragmented, M = missing, n = number of orthologues in comparison.

**Figure 2.**
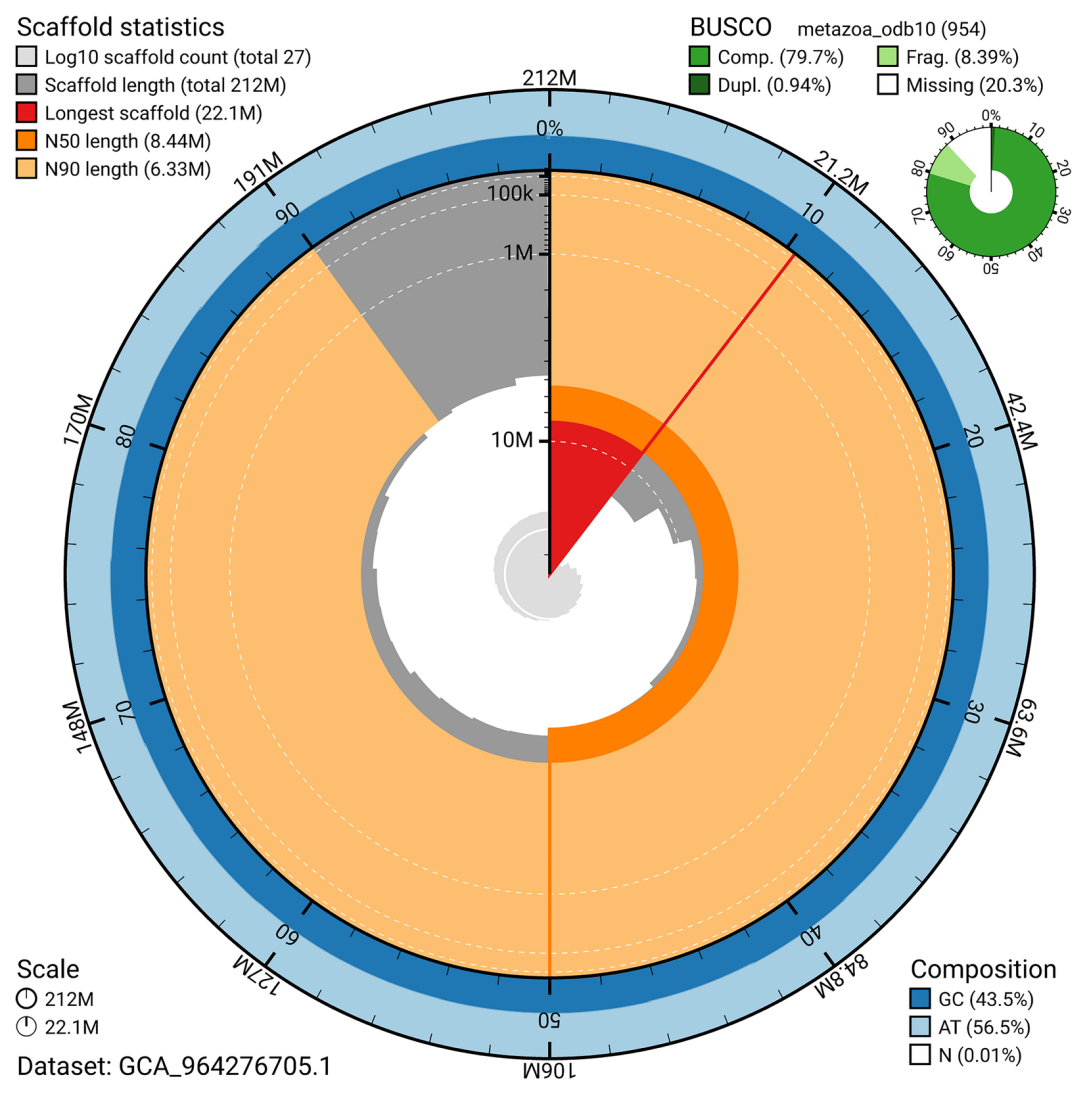
Genome assembly of *Phakellia ventilabrum*, odPhaVent3.1: metrics. The BlobToolKit snail plot provides an overview of assembly metrics and BUSCO gene completeness. The circumference represents the length of the whole genome sequence, and the main plot is divided into 1,000 bins around the circumference. The outermost blue tracks display the distribution of GC, AT, and N percentages across the bins. Scaffolds are arranged clockwise from longest to shortest and are depicted in dark grey. The longest scaffold is indicated by the red arc, and the deeper orange and pale orange arcs represent the N50 and N90 lengths. A light grey spiral at the centre shows the cumulative scaffold count on a logarithmic scale. A summary of complete, fragmented, duplicated, and missing BUSCO genes in the metazoa_odb10 set is presented at the top right. An interactive version of this figure is available at https://blobtoolkit.genomehubs.org/view/GCA_964276705.1/dataset/GCA_964276705.1/snail.

**Figure 3.**
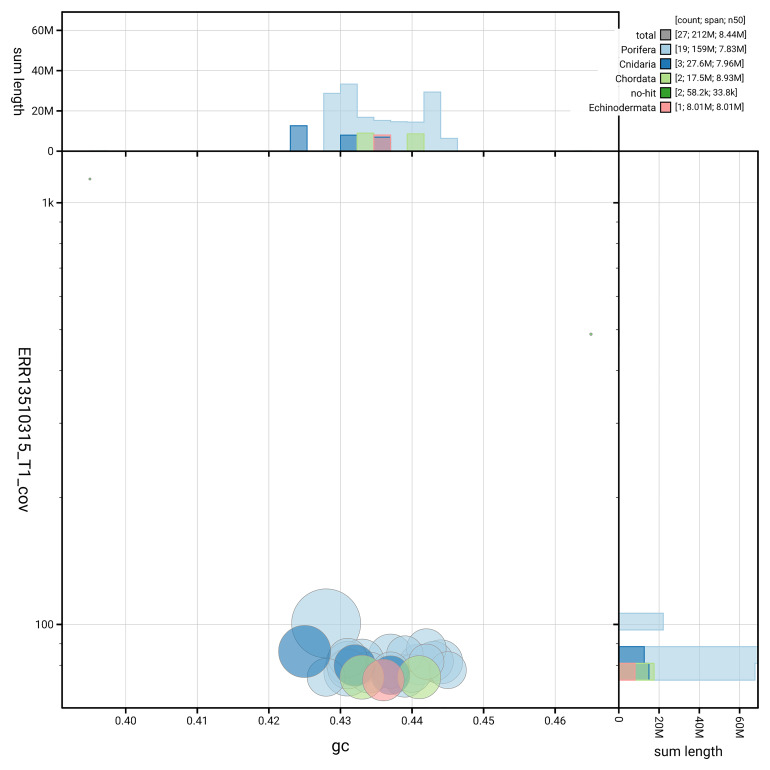
Genome assembly of *Phakellia ventilabrum*, odPhaVent3.1: BlobToolKit GC-coverage plot. Blob plot showing sequence coverage (vertical axis) and GC content (horizontal axis). The circles represent scaffolds, with the size proportional to scaffold length and the colour representing phylum membership. The histograms along the axes display the total length of sequences distributed across different levels of coverage and GC content. An interactive version of this figure is available at https://blobtoolkit.genomehubs.org/view/GCA_964276705.1/dataset/GCA_964276705.1/blob.

The snail plot in Figure 2 provides a summary of the assembly statistics, indicating the distribution of scaffold lengths and other assembly metrics. Figure 3 shows the distribution of scaffolds by GC proportion and coverage.

**Figure 4.**
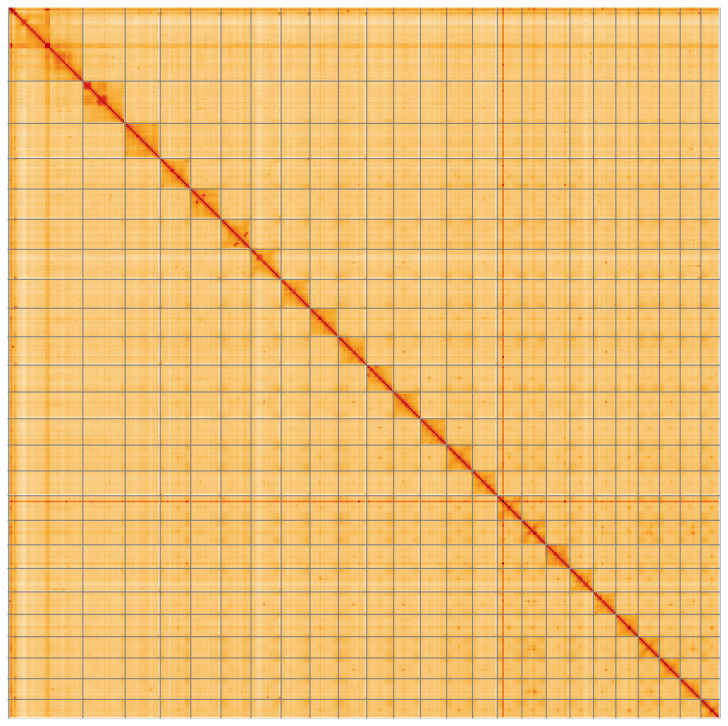
Genome assembly of *Phakellia ventilabrum*: Hi-C contact map of the odPhaVent3.1 assembly, visualised using HiGlass. Chromosomes are shown in order of size from left to right and top to bottom. An interactive version of this figure may be viewed at https://genome-note-higlass.tol.sanger.ac.uk/l/?d=OdUSwO9xSWKqKrRfqJkbRg.

Most of the assembly sequence (99.98%) was assigned to 25 chromosomal-level scaffolds. These chromosome-level scaffolds, confirmed by Hi-C data, are named according to size ( Figure 4; Table 3).

**Table 3.** Chromosomal pseudomolecules in the genome assembly of *Phakellia ventilabrum*, odPhaVent3.

INSDC accession	Name	Length (Mb)	GC%
OZ194358.1	1	22.06	43
OZ194359.1	2	12.61	42.5
OZ194360.1	3	10.43	43
OZ194361.1	4	9.1	43.5
OZ194362.1	5	8.98	43.5
OZ194363.1	6	8.97	44.5
OZ194364.1	7	8.93	43.5
OZ194365.1	8	8.6	44
OZ194366.1	9	8.49	43
OZ194367.1	10	8.44	44
OZ194368.1	11	8.01	43.5
OZ194369.1	12	7.96	43
OZ194370.1	13	7.83	43.5
OZ194371.1	14	7.7	44
OZ194372.1	15	7.37	43
OZ194373.1	16	7.32	44.5
OZ194374.1	17	7.29	44
OZ194375.1	18	7.03	43
OZ194376.1	19	6.99	43.5
OZ194377.1	20	6.68	44
OZ194378.1	21	6.66	43
OZ194379.1	22	6.33	44.5
OZ194380.1	23	6.17	44
OZ194381.1	24	6.17	43.5
OZ194382.1	25	5.77	44
OZ194383.1	MT	0.02	39.5

The mitochondrial genome was also assembled. This sequence is included as a contig in the multifasta file of the genome submission and as a standalone record in GenBank.

### Assembly quality metrics

The primary haplotype has a QV of 63.4, and the combined primary and alternate assemblies achieve an estimated QV of 62.3. BUSCO v.5.5.0 analysis using the metazoa_odb10 reference set ( *n* = 954) indicated a completeness score of 79.7% (single = 78.7%, duplicated = 0.9%).

### Metagenome report

**Figure 5.**
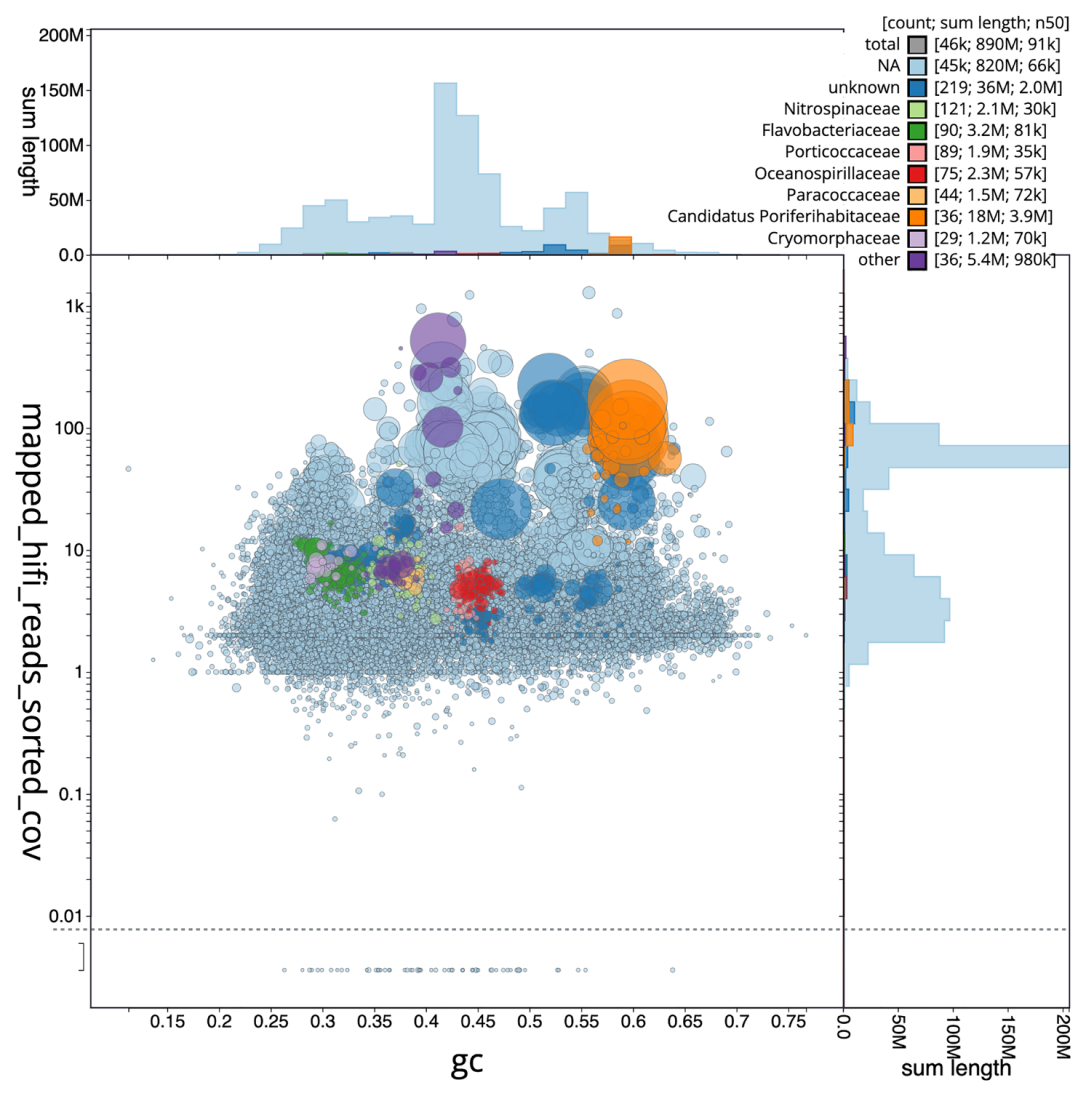
Blob plot of base coverage mapped against GC proportion for sequences in the *Phakellia ventilabrum* metagenome. Binned contigs are coloured by family. Circles are sized in proportion to sequence length on a square-root scale, ranging from 510 to 6 879 066. Histograms show the distribution of sequence length sum along each axis. An interactive version of this figure may be viewed here.

**Figure 6.**
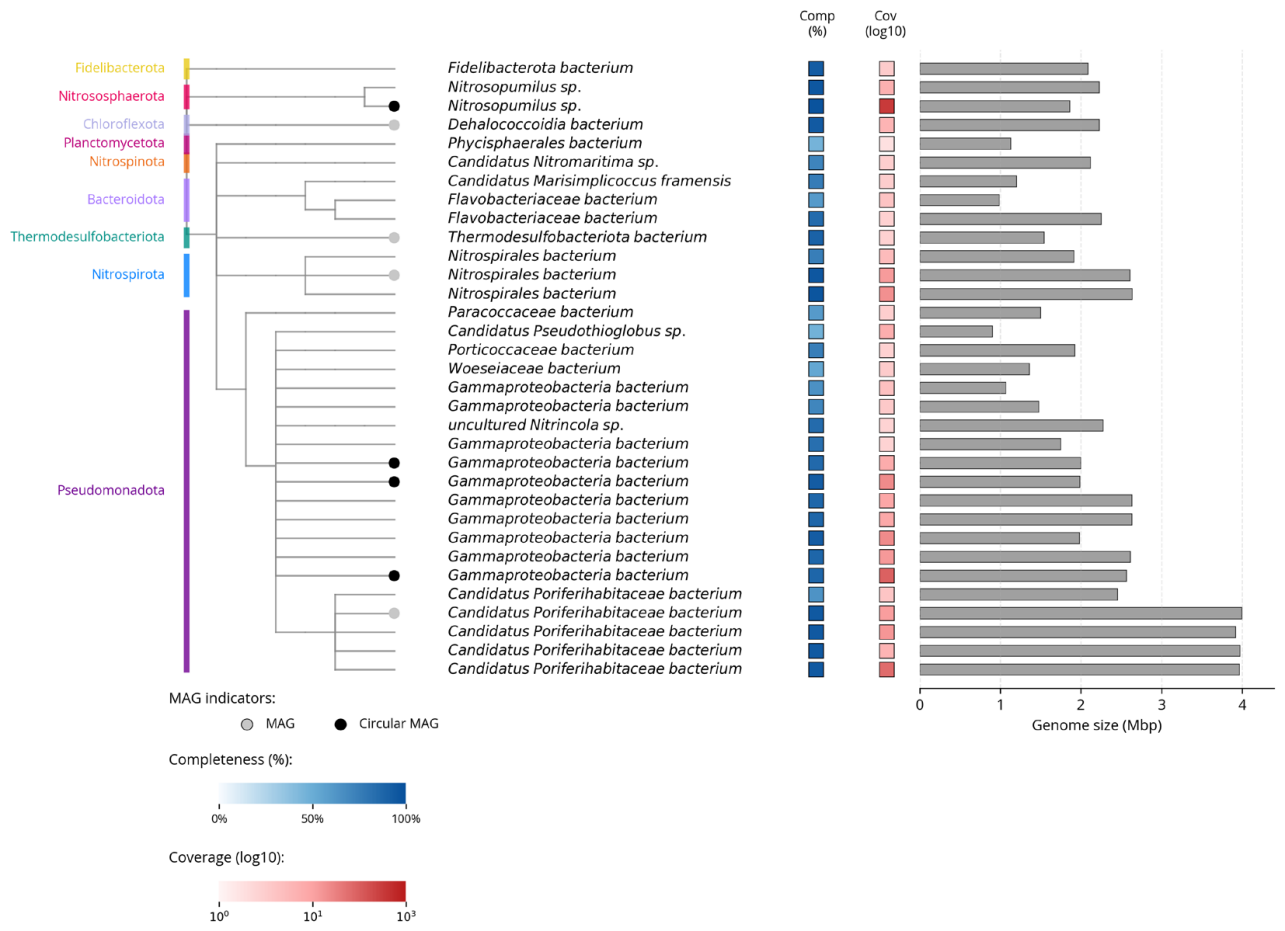
Taxonomic tree based on taxonomic classifications of metagenome bins, constructed using ete3. Colours indicate phylum-level taxonomy. Tracks show genome completeness (blue), sequencing coverage (red, log10), and genome size (grey bars, Mbp). High-quality MAGs are marked with grey circles; fully circularized MAGs in black.

We recovered 33 bins from the metagenome assembly ( Figure 5), of which eight met the criteria for MAGs, including four fully circularised genomes. The recovered bins represented 9 bacterial and archaeal phyla, with genome sizes ranging from 0.90 to 4.00 Mbp (mean: 2.17 ± 0.83 Mbp). Mean completeness was 81.9% (± 15.1%) with 2.7% (± 2.8%) contamination. Figure 6 summarises the taxa and quality of the metagenome bins. The full per-bin table of taxa and quality metrics for the metagenome bins is available on Zenodo.

### Genome annotation report

The *Phakellia ventilabrum* genome assembly (GCA_964276705.1) was annotated by Ensembl at the European Bioinformatics Institute (EBI). This annotation includes 38 110 transcribed mRNAs from 21 622 protein-coding and 5 189 non-coding genes. The average transcript length is 7 147.00 bp, with an average of 1.42 coding transcripts per gene and 6.91 exons per transcript. For further information about the annotation, please refer to the Ensembl annotation page.

## Methods

### Sample acquisition

A specimen of *P. ventilabrum* (specimen ID GHC0000295, ToLID odPhaVent3; Figure 1) was collected on 2018-08-12 by Remotely Operated Vehicle (ROV) *Ægir6000*, event GS2018108-70-ROV-50, during the GS2018108 cruise onboard research vessel *G.O. Sars*. It was collected in the Norwegian Sea at a water depth of 218 m in the Stjernsund (latitude 70.2632°N, longitude 22.4744°E). The specimen was subsampled, flash-frozen and stored at –80 °C at GEOMAR until processing for genome sequencing. The specimen was collected by Kathrin Busch (Geomar) and identified by Hans Tore Rapp (UiB, Norway).

### Nucleic acid extraction

The workflow for high molecular weight (HMW) DNA extraction at the Wellcome Sanger Institute (WSI) Tree of Life Core Laboratory includes a sequence of procedures: sample preparation and homogenisation, DNA extraction, fragmentation and purification. Detailed protocols are available on protocols.io ( Denton *et al.*, 2023). The odPhaVent3 sample was prepared for DNA extraction by weighing and dissecting it on dry ice ( Jay *et al.*, 2023). Prior to DNA extraction, the sponge sample was bathed in “L buffer” (10 mM Tris, pH 7.6, 100 mM EDTA, 20 mM NaCl), minced into small pieces using a scalpel and the cellular interior separated from the mesohyl using forceps ( Lopez, 2022). HMW DNA was extracted using the Automated MagAttract v2 protocol ( Oatley *et al.*, 2023a). DNA was sheared into an average fragment size of 12–20 kb in a Megaruptor 3 system ( Bates *et al.*, 2023). Sheared DNA was purified by solid-phase reversible immobilisation, using AMPure PB beads to eliminate shorter fragments and concentrate the DNA ( Oatley *et al.*, 2023b). The concentration of the sheared and purified DNA was assessed using a Nanodrop spectrophotometer and Qubit Fluorometer using the Qubit dsDNA High Sensitivity Assay kit. Fragment size distribution was evaluated by running the sample on the FemtoPulse system.

RNA was also extracted from somatic tissue of odPhaVent3 in the Tree of Life Laboratory at the WSI using the RNA Extraction: Automated MagMax™ *mir*Vana protocol ( do Amaral *et al.*, 2023). The RNA concentration was assessed using a Nanodrop spectrophotometer and a Qubit Fluorometer using the Qubit RNA Broad-Range Assay kit. Analysis of the integrity of the RNA was done using the Agilent RNA 6000 Pico Kit and Eukaryotic Total RNA assay.

### Sequencing

Pacific Biosciences HiFi circular consensus DNA sequencing libraries were constructed according to the manufacturers’ instructions. DNA sequencing was performed by the Scientific Operations core at the WSI on Pacific Biosciences Revio. Tissue from the somatic tissue of the odPhaVent3 sample was processed for Hi-C sequencing at the WSI Scientific Operations core, using the Arima-HiC v2 kit.and sequenced on the Illumina NovaSeq X instrument. Poly(A) RNA-Seq libraries were constructed using the NEB Ultra II RNA Library Prep kit, following the manufacturer’s instructions. RNA sequencing was performed on the Illumina NovaSeq X instrument.

## Genome assembly, curation and evaluation

### Assembly

Prior to assembly of the PacBio HiFi reads, a database of *k*-mer counts ( *k* = 31) was generated from the filtered reads using FastK. GenomeScope2 ( Ranallo-Benavidez *et al.*, 2020) was used to analyse the *k*-mer frequency distributions, providing estimates of genome size, heterozygosity, and repeat content.

The HiFi reads were assembled using Hifiasm ( Cheng *et al.*, 2021) with the --primary option. Haplotypic duplications were identified and removed using purge_dups ( Guan *et al.*, 2020). The Hi-C reads were mapped to the primary contigs using bwa-mem2 ( Vasimuddin *et al.*, 2019). The contigs were further scaffolded using the provided Hi-C data ( Rao *et al.*, 2014) in YaHS ( Zhou *et al.*, 2023) using the --break option for handling potential misassemblies. The scaffolded assemblies were evaluated using Gfastats ( Formenti *et al.*, 2022), BUSCO ( Manni *et al.*, 2021) and MERQURY.FK ( Rhie *et al.*, 2020).

The mitochondrial genome was assembled using MitoHiFi ( Uliano-Silva *et al.*, 2023), which runs MitoFinder ( Allio *et al.*, 2020) and uses these annotations to select the final mitochondrial contig and to ensure the general quality of the sequence.

### Assembly curation

The assembly was decontaminated using the Assembly Screen for Cobionts and Contaminants (ASCC) pipeline. Flat files and maps used in curation were generated via the TreeVal pipeline ( Pointon *et al.*, 2023). Manual curation was conducted primarily in PretextView ( Harry, 2022) and HiGlass ( Kerpedjiev *et al.*, 2018), with additional insights provided by JBrowse2 ( Diesh *et al.*, 2023). Scaffolds were visually inspected and corrected as described by Howe *et al*. (2021). Any identified contamination, missed joins, and mis-joins were amended, and duplicate sequences were tagged and removed. The curation process is documented at https://gitlab.com/wtsi-grit/rapid-curation.

### Taxonomic verification

Sequences of type material have not been available for taxonomic validation. Therefore, we reconstructed phylogenetic trees with the mitochondrial cytochrome oxidase subunit 1 (Folmer-region) and the C-region of the nuclear long ribosomal subunit (28S-C) for all sequences currently published for this genus. Sequences were aligned using MAFFT v7.450 ( Katoh & Standley, 2013), and Maximum Likelihood trees were reconstructed using RAxML 8 (GTR+GAMMA+ I model, Stamatakis, 2014). In both analyses the current sample was part of a monophyletic group of *P. ventilabrum*, comprising other, well-identified samples (e.g., HQ379409 (CO1, identical), or HQ379197 (28S, 1 bp different), both Morrow *et al.* (2012)). In addition, to validate the taxonomic identity of the sequenced specimen, the cox1 gene sequence (animal DNA barcode) of a previously sequenced *P. ventilabrum* specimen spanning 658 bp was retrieved from NCBI (accession: HQ379409.1). This sequence was aligned against the scaffold identified as the mitochondrial genome of the specimen used for assembling the reference genome (NCBI accession: OZ194383.1), using the software Geneious Prime v.2025.2.1 under default parameters. The barcode sequence was aligned from position 14,135 to position 14,793 of the new mitochondrial genome assembly. The alignment showed a 100% sequence identity, thereby providing molecular confirmation that indeed the assembled individual belonged to the species *P. ventilabrum*.

### Assembly quality assessment

The Merqury.FK tool ( Rhie *et al.*, 2020), run in a Singularity container ( Kurtzer *et al.*, 2017), was used to evaluate the assembly quality of the primary and alternate haplotypes using the *k*-mer databases ( *k* = 31) that were computed prior to genome assembly.

A Hi-C contact map was produced for the final version of the assembly. The Hi-C reads were aligned using bwa-mem2 ( Vasimuddin *et al.*, 2019) and the alignment files were combined using SAMtools ( Danecek *et al.*, 2021). The Hi-C alignments were converted into a contact map using BEDTools ( Quinlan & Hall, 2010) and the Cooler tool suite ( Abdennur & Mirny, 2020).

The genome was analysed using the BlobToolkit pipeline, a Nextflow port of the earlier Snakemake version ( Challis *et al.*, 2020). The pipeline aligns PacBio reads using SAMtools and minimap2 ( Li, 2018) and generates coverage tracks for regions of fixed size. It runs BUSCO ( Manni *et al.*, 2021) using lineages identified from the NCBI taxonomy ( Schoch *et al.*, 2020). For the three domain-level lineages, BUSCO genes are aligned to the UniProt Reference Proteomes ( Bateman *et al.*, 2023) using DIAMOND blastp ( Buchfink *et al.*, 2021). The genome is divided into chunks according to the density of the BUSCO genes from the closest taxonomic lineage, and each chunk is aligned to the UniProt Reference Proteomes using DIAMOND blastx. Genome sequences without a hit are split using seqtk and aligned to the NT database with blastn ( Altschul *et al.*, 1990). The blobtools suite consolidates all outputs into a blobdir for visualisation. The BlobToolKit pipeline was developed using nf-core tooling ( Ewels *et al.*, 2020) and MultiQC ( Ewels *et al.*, 2016), with containerisation through Docker ( Merkel, 2014) and Singularity ( Kurtzer *et al.*, 2017).

Table 4 contains a list of relevant software tool versions and sources.

**Table 4.** Software tools: versions and sources.

Software tool	Version	Source
BEDTools	2.30.0	https://github.com/arq5x/bedtools2
bin3C	0.3.3	https://github.com/cerebis/bin3C
BLAST	2.14.0	ftp://ftp.ncbi.nlm.nih.gov/blast/executables/blast+/
BlobToolKit	4.3.9	https://github.com/blobtoolkit/blobtoolkit
BUSCO	5.5.0	https://gitlab.com/ezlab/busco
bwa-mem2	2.2.1	https://github.com/bwa-mem2/bwa-mem2
CheckM	1.2.1	https://github.com/Ecogenomics/CheckM
Cooler	0.8.11	https://github.com/open2c/cooler
DIAMOND	2.1.8	https://github.com/bbuchfink/diamond
dRep	3.4.0	https://github.com/MrOlm/drep
fasta_windows	0.2.4	https://github.com/tolkit/fasta_windows
FastK	427104ea91c78c3b8b8b49f1a7d6bbeaa869ba1c	https://github.com/thegenemyers/FASTK
Gfastats	1.3.6	https://github.com/vgl-hub/gfastats
GTDB-TK	2.3.2	https://github.com/Ecogenomics/GTDBTk
Hifiasm	0.19.8-r603	https://github.com/chhylp123/hifiasm
HiGlass	44086069ee7d4d3f6f3f0012569789ec138f42b84 aa44357826c0b6753eb28de	https://github.com/higlass/higlass
MAGScoT	1.0.0	https://github.com/ikmb/MAGScoT
MaxBin	2.7	https://sourceforge.net/projects/maxbin/
MerquryFK	d00d98157618f4e8d1a9190026b19b471055b2 2e	https://github.com/thegenemyers/MERQURY.FK
MetaBat2	2.15-15-gd6ea400	https://bitbucket.org/berkeleylab/metabat/src/master/
metaMDBG	-	https://github.com/GaetanBenoitDev/metaMDBG
Minimap2	2.24-r1122	https://github.com/lh3/minimap2
MitoHiFi	2	https://github.com/marcelauliano/MitoHiFi
MultiQC	1.14, 1.17, and 1.18	https://github.com/MultiQC/MultiQC
NCBI Datasets	15.12.0	https://github.com/ncbi/datasets
Nextflow	23.10.0	https://github.com/nextflow-io/nextflow
PretextView	0.2	https://github.com/sanger-tol/PretextView
PROKKA	1.14.5	https://github.com/vdejager/prokka
purge_dups	1.2.3	https://github.com/dfguan/purge_dups
samtools	1.19.2	https://github.com/samtools/samtools
sanger-tol/ascc	-	https://github.com/sanger-tol/ascc
sanger-tol/blobtoolkit	0.6.0	https://github.com/sanger-tol/blobtoolkit
Seqtk	1.3	https://github.com/lh3/seqtk
Singularity	3.9.0	https://github.com/sylabs/singularity
TreeVal	1.2.0	https://github.com/sanger-tol/treeval
YaHS	1.1a.2	https://github.com/c-zhou/yahs

## Metagenome assembly

The metagenome assembly was generated using metaMDBG ( Benoit *et al.*, 2024) and binned using MetaBAT2 ( Kang *et al.*, 2019), MaxBin ( Wu *et al.*, 2014), bin3C ( DeMaere & Darling, 2019), and MetaTOR. The resulting bin sets of each binning algorithm were optimised and refined using MAGScoT ( Rühlemann *et al.*, 2022). PROKKA ( Seemann, 2014) was used to identify tRNAs and rRNAs in each bin, CheckM ( Parks *et al.*, 2015) (checkM_DB release 2015-01-16) was used to assess bin completeness/contamination, and GTDB-TK ( Chaumeil *et al.*, 2022) (GTDB release 214) was used to taxonomically classify bins. Taxonomic replicate bins were identified using dRep ( Olm *et al.*, 2017) with default settings (95% ANI threshold). The final bin set was filtered for bacteria and archaea. All bins were assessed for quality and categorised as metagenome-assembled genomes (MAGs) if they met the following criteria: contamination ≤ 5%, presence of 5S, 16S, and 23S rRNA genes, at least 18 unique tRNAs, and either ≥ 90% completeness or ≥ 50% completeness with fully circularised chromosomes. Bins that did not meet these thresholds, or were identified as taxonomic replicates of MAGs, were retained as ‘binned metagenomes’ provided they had ≥ 50% completeness and ≤ 10% contamination. A taxonomic tree of the bins was constructed from NCBI classifications using ete3 ( Huerta-Cepas *et al.*, 2016) and visualised with matplotlib. Software tool versions and sources are given in Table 4.

## Wellcome Sanger Institute – Legal and Governance

The materials that have contributed to this genome note have been supplied by a Tree of Life collaborator. The Wellcome Sanger Institute employs a process whereby due diligence is carried out proportionate to the nature of the materials themselves, and the circumstances under which they have been/are to be collected and provided for use. The purpose of this is to address and mitigate any potential legal and/or ethical implications of receipt and use of the materials as part of the research project, and to ensure that in doing so we align with best practice wherever possible. The overarching areas of consideration are:

- Ethical review of provenance and sourcing of the material
- Legality of collection, transfer and use (national and international)

Each transfer of samples is undertaken according to a Research Collaboration Agreement or Material Transfer Agreement entered into by the Tree of Life collaborator, Genome Research Limited (operating as the Wellcome Sanger Institute) and in some circumstances other Tree of Life collaborators.

## Data Availability

European Nucleotide Archive: *Phakellia ventilabrum*. Accession number PRJEB78906; https://identifiers.org/ena.embl/PRJEB78906. The genome sequence is released openly for reuse. The *Phakellia ventilabrum* genome sequencing initiative is part of the Aquatic Symbiosis Genomics (ASG) project ( https://www.ebi.ac.uk/ena/browser/view/PRJEB43743). All raw sequence data and the assembly have been deposited in INSDC databases. The genome will be annotated using available RNA-Seq data and presented through the Ensembl pipeline at the European Bioinformatics Institute. Raw data and assembly accession identifiers are reported in Table 1 and Table 2.

## Author information

Contributors are listed at the following links:

- Wellcome Sanger Institute Tree of Life Management, Samples and Laboratory team
- Wellcome Sanger Institute Scientific Operations – Sequencing Operations
- Wellcome Sanger Institute Tree of Life Core Informatics team
- Tree of Life Core Informatics collective
- European Bioinformatics Institute ASG Data Portal team
- Wellcome Sanger Institute/Aquatic Symbiosis Genomics Project Leadership

## References

[ref-1] Abdennur N Mirny LA : Cooler: scalable storage for Hi-C data and other genomically labeled arrays. *Bioinformatics.* 2020;36(1):311–316. 10.1093/bioinformatics/btz540 31290943 PMC8205516

[ref-2] Allio R Schomaker-Bastos A Romiguier J : MitoFinder: efficient automated large-scale extraction of mitogenomic data in target enrichment phylogenomics. *Mol Ecol Resour.* 2020;20(4):892–905. 10.1111/1755-0998.13160 32243090 PMC7497042

[ref-3] Altschul SF Gish W Miller W : Basic Local Alignment Search Tool. *J Mol Biol.* 1990;215(3):403–410. 10.1016/S0022-2836(05)80360-2 2231712

[ref-4] Bateman A Martin MJ Orchard S : UniProt: the Universal Protein Knowledgebase in 2023. *Nucleic Acids Res.* 2023;51(D1):D523–D531. 10.1093/nar/gkac1052 36408920 PMC9825514

[ref-5] Bates A Clayton-Lucey I Howard C : Sanger Tree of Life HMW DNA fragmentation: diagenode megaruptor ^®^3 for LI PacBio. *protocols.io.* 2023. 10.17504/protocols.io.81wgbxzq3lpk/v1

[ref-6] Benoit G Raguideau S James R : High-quality metagenome assembly from long accurate reads with metaMDBG. *Nat Biotechnol.* 2024:42(9):1378–1383. 10.1038/s41587-023-01983-6 38168989 PMC11392814

[ref-7] Buchfink B Reuter K Drost HG : Sensitive protein alignments at Tree-of-Life scale using DIAMOND. *Nat Methods.* 2021;18(4):366–368. 10.1038/s41592-021-01101-x 33828273 PMC8026399

[ref-8] Busch K Slaby BM Bach W : Biodiversity, environmental drivers, and sustainability of the global deep-sea sponge microbiome. *Nat Commun.* 2022;13(1): 5160. 10.1038/s41467-022-32684-4 36056000 PMC9440067

[ref-9] Challis R Richards E Rajan J : BlobToolKit – interactive quality assessment of genome assemblies. *G3 (Bethesda).* 2020;10(4):1361–1374. 10.1534/g3.119.400908 32071071 PMC7144090

[ref-10] Chaumeil PA Mussig AJ Hugenholtz P : GTDB-Tk v2: memory friendly classification with the genome taxonomy database. *Bioinformatics.* 2022;38(23):5315–5316. 10.1093/bioinformatics/btac672 36218463 PMC9710552

[ref-11] Cheng H Concepcion GT Feng X : Haplotype-resolved *de novo* assembly using phased assembly graphs with hifiasm. *Nat Methods.* 2021;18(2):170–175. 10.1038/s41592-020-01056-5 33526886 PMC7961889

[ref-12] Danecek P Bonfield JK Liddle J : Twelve years of SAMtools and BCFtools. *GigaScience.* 2021;10(2): giab008. 10.1093/gigascience/giab008 33590861 PMC7931819

[ref-14] DeMaere MZ Darling AE : bin3C: exploiting Hi-C sequencing data to accurately resolve metagenome-assembled genomes. *Genome Biol.* 2019;20(1): 46. 10.1186/s13059-019-1643-1 30808380 PMC6391755

[ref-15] Denton A Yatsenko H Jay J : Sanger Tree of Life wet laboratory protocol collection. *protocols.io.* 2023. 10.17504/protocols.io.8epv5xxy6g1b/v1

[ref-13] de Voogd NJ Alvarez B Boury-Esnault N : World Porifera database.2024. 10.14284/359

[ref-16] Diesh C Stevens GJ Xie P : JBrowse 2: a modular genome browser with views of synteny and structural variation. *Genome Biol.* 2023;24(1): 74. 10.1186/s13059-023-02914-z 37069644 PMC10108523

[ref-17] Díez-Vives C Riesgo A : High compositional and functional similarity in the microbiome of deep-sea sponges. *ISME J.* 2024;18(1): wrad030. 10.1093/ismejo/wrad030 38365260 PMC10837836

[ref-18] do Amaral RJV Bates A Denton A : Sanger Tree of Life RNA extraction: automated MagMax ^TM^ mirVana. *protocols.io.* 2023. 10.17504/protocols.io.6qpvr36n3vmk/v1

[ref-19] Ewels P Magnusson M Lundin S : MultiQC: summarize analysis results for multiple tools and samples in a single report. *Bioinformatics.* 2016;32(19):3047–3048. 10.1093/bioinformatics/btw354 27312411 PMC5039924

[ref-20] Ewels PA Peltzer A Fillinger S : The nf-core framework for community-curated bioinformatics pipelines. *Nat Biotechnol.* 2020;38(3):276–278. 10.1038/s41587-020-0439-x 32055031

[ref-21] Formenti G Abueg L Brajuka A : Gfastats: conversion, evaluation and manipulation of genome sequences using assembly graphs. *Bioinformatics.* 2022;38(17):4214–4216. 10.1093/bioinformatics/btac460 35799367 PMC9438950

[ref-22] Gallego R Arias MB Corral-Lou A : North Atlantic deep-sea benthic biodiversity unveiled through sponge natural sampler DNA. *Commun Biol.* 2024;7(1): 1015. 10.1038/s42003-024-06695-4 39160260 PMC11333605

[ref-23] Guan D McCarthy SA Wood J : Identifying and removing haplotypic duplication in primary genome assemblies. *Bioinformatics.* 2020;36(9):2896–2898. 10.1093/bioinformatics/btaa025 31971576 PMC7203741

[ref-24] Harry E : PretextView (Paired REad TEXTure Viewer): a desktop application for viewing pretext contact maps.2022. Reference Source

[ref-25] Hentschel U Piel J Degnan SM : Genomic insights into the marine sponge microbiome. *Nat Rev Microbiol.* 2012;10(9):641–654. 10.1038/nrmicro2839 22842661

[ref-26] Howe K Chow W Collins J : Significantly improving the quality of genome assemblies through curation. *GigaScience.* 2021;10(1): giaa153. 10.1093/gigascience/giaa153 33420778 PMC7794651

[ref-27] Huerta-Cepas J Serra F Bork P : ETE 3: reconstruction, analysis, and visualization of phylogenomic data. *Mol Biol Evol.* 2016;33(6):1635–38. 10.1093/molbev/msw046 26921390 PMC4868116

[ref-28] Jay J Yatsenko H Narváez-Gómez JP : Sanger Tree of Life sample preparation: triage and dissection. *protocols.io.* 2023. 10.17504/protocols.io.x54v9prmqg3e/v1

[ref-29] Kang DD Li F Kirton E : MetaBAT 2: an adaptive binning algorithm for robust and efficient genome reconstruction from metagenome assemblies. *PeerJ.* 2019;7: e7359. 10.7717/peerj.7359 31388474 PMC6662567

[ref-30] Katoh K Standley DM : MAFFT multiple sequence alignment software version 7: improvements in performance and usability. *Mol Biol Evol.* 2013;30(4):772–780. 10.1093/molbev/mst010 23329690 PMC3603318

[ref-31] Kerpedjiev P Abdennur N Lekschas F : HiGlass: web-based visual exploration and analysis of genome interaction maps. *Genome Biol.* 2018;19(1): 125. 10.1186/s13059-018-1486-1 30143029 PMC6109259

[ref-32] Koutsouveli V Balgoma D Checa A : Oogenesis and lipid metabolism in the deep-sea sponge *Phakellia ventilabrum* (Linnaeus, 1767). *Sci Rep.* 2022;12(1): 6317. 10.1038/s41598-022-10058-6 35428825 PMC9012834

[ref-33] Kurtzer GM Sochat V Bauer MW : Singularity: scientific containers for mobility of compute. *PLoS One.* 2017;12(5): e0177459. 10.1371/journal.pone.0177459 28494014 PMC5426675

[ref-34] Li H : Minimap2: pairwise alignment for nucleotide sequences. *Bioinformatics.* 2018;34(18):3094–3100. 10.1093/bioinformatics/bty191 29750242 PMC6137996

[ref-35] Lopez J : Squeeze-enrichment of intact cells (eukaryotic and prokaryotic) from marine sponge tissues prior to routine DNA extraction. *protocols.io.* 2022. 10.17504/protocols.io.n92ldzj4ov5b/v1

[ref-36] Maldonado M Aguilar R Bannister RJ : Sponge grounds as key marine habitats: a synthetic review of types, structure, functional roles, and conservation concerns. In: *Marine animal forests: The ecology of benthic biodiversity hotspots*.2017;145–183. 10.1007/978-3-319-17001-5_24-1

[ref-37] Manni M Berkeley MR Seppey M : BUSCO update: novel and streamlined workflows along with broader and deeper phylogenetic coverage for scoring of eukaryotic, prokaryotic, and viral genomes. *Mol Biol Evol.* 2021;38(10):4647–4654. 10.1093/molbev/msab199 34320186 PMC8476166

[ref-38] Merkel D : Docker: lightweight Linux containers for consistent development and deployment. *Linux J.* 2014;2014(239): 2. Reference Source

[ref-39] Mc Cartney AM Formenti G Mouton A : The European reference genome atlas: piloting a decentralised approach to equitable biodiversity genomics. *NPJ Biodivers.* 2024;3(1): 28. 10.1038/s44185-024-00054-6 39289538 PMC11408602

[ref-40] Morrow CC Picton BE Erpenbeck D : Congruence between nuclear and mitochondrial genes in demospongiae: a new hypothesis for relationships within the G4 clade (Porifera: Demospongiae). *Mol Phylogenet Evol.* 2012;62(1):174–190. 10.1016/j.ympev.2011.09.016 22001855

[ref-41] Neave EF Cai W Arias MB : Trapped DNA fragments in marine sponge specimens unveil North Atlantic deep-sea fish diversity. *Proc Biol Sci.* 2023;290(2005): 20230771. 10.1098/rspb.2023.0771 37644836 PMC10465980

[ref-42] Oatley G Denton A Howard C : Sanger Tree of Life HMW DNA extraction: automated MagAttract v.2. *protocols.io.* 2023a. 10.17504/protocols.io.kxygx3y4dg8j/v1

[ref-43] Oatley G Sampaio F Howard C : Sanger Tree of Life fragmented DNA clean up: automated SPRI. *protocols.io.* 2023b. 10.17504/protocols.io.q26g7p1wkgwz/v1

[ref-44] Olm MR Brown CT Brooks B : dRep: a tool for fast and accurate genomic comparisons that enables improved genome recovery from metagenomes through de-replication. *ISME J.* 2017;11(12):2864–2868. 10.1038/ismej.2017.126 28742071 PMC5702732

[ref-45] Parks DH Imelfort M Skennerton CT : CheckM: assessing the quality of microbial genomes recovered from isolates, single cells, and metagenomes. *Genome Res.* 2015;25(7):1043–55. 10.1101/gr.186072.114 25977477 PMC4484387

[ref-46] Pointon DL Eagles W Sims Y : sanger-tol/treeval v1.0.0 – Ancient Atlantis.2023. 10.5281/zenodo.10047653

[ref-47] Prado E Rodríguez-Basalo A Cobo A : 3D fine-scale terrain variables from underwater photogrammetry: a new approach to benthic microhabitat modeling in a circalittoral Rocky shelf. *Remote Sens.* 2020;12(15): 2466. 10.3390/RS12152466

[ref-48] Quinlan AR Hall IM : BEDTools: a flexible suite of utilities for comparing genomic features. *Bioinformatics.* 2010;26(6):841–842. 10.1093/bioinformatics/btq033 20110278 PMC2832824

[ref-49] Ranallo-Benavidez TR Jaron KS Schatz MC : GenomeScope 2.0 and Smudgeplot for reference-free profiling of polyploid genomes. *Nat Commun.* 2020;11(1): 1432. 10.1038/s41467-020-14998-3 32188846 PMC7080791

[ref-50] Rao SSP Huntley MH Durand NC : A 3D map of the human genome at kilobase resolution reveals principles of chromatin looping. *Cell.* 2014;159(7):1665–1680. 10.1016/j.cell.2014.11.021 25497547 PMC5635824

[ref-51] Redmond NE Morrow CC Thacker RW : Phylogeny and systematics of demospongiae in light of new small-subunit ribosomal DNA (18S) sequences. *Integr Comp Biol.* 2013;53(3):388–415. 10.1093/icb/ict078 23793549

[ref-52] Rhie A Walenz BP Koren S : Merqury: reference-free quality, completeness, and phasing assessment for genome assemblies. *Genome Biol.* 2020;21(1): 245. 10.1186/s13059-020-02134-9 32928274 PMC7488777

[ref-53] Rühlemann MC Wacker EM Ellinghaus D : MAGScoT: a fast, lightweight and accurate bin-refinement tool. *Bioinformatics.* 2022;38(24):5430–5433. 10.1093/bioinformatics/btac694 36264141 PMC9750101

[ref-54] Sánchez F Serrano A Ballesteros MG : Photogrammetric quantitative study of habitat and benthic communities of deep Cantabrian Sea hard grounds. *Cont Shelf Res.* 2009;29(8):1174–1188. 10.1016/j.csr.2009.01.004

[ref-55] Schoch CL Ciufo S Domrachev M : NCBI taxonomy: a comprehensive update on curation, resources and tools. *Database (Oxford).* 2020;2020: baaa062. 10.1093/database/baaa062 32761142 PMC7408187

[ref-56] Seemann T : Prokka: rapid prokaryotic genome annotation. *Bioinformatics.* 2014;30(14):2068–2069. 10.1093/bioinformatics/btu153 24642063

[ref-57] Stamatakis A : RAxML version 8: a tool for phylogenetic analysis and post-analysis of large phylogenies. *Bioinformatics.* 2014;30(9):1312–1313. 10.1093/bioinformatics/btu033 24451623 PMC3998144

[ref-58] Taboada S Ríos P Mitchell A : Genetic diversity, gene flow and hybridization in fan-shaped sponges ( *Phakellia* spp.) in the North-East Atlantic deep sea. *Deep Sea Research Part I: Oceanographic Research Papers.* 2022;181: 103685. 10.1016/J.DSR.2021.103685

[ref-59] Taboada S Whiting C Wang S : Long distance dispersal and oceanographic fronts shape the connectivity of the keystone sponge *Phakellia ventilabrum* in the deep northeast Atlantic. *Front Mar Sci.* 2023;10: 1177106. 10.3389/fmars.2023.1177106

[ref-60] Thomas T Moitinho-Silva L Lurgi M : Diversity, structure and convergent evolution of the global sponge microbiome. *Nat Commun.* 2016;7(1): 11870. 10.1038/ncomms11870 27306690 PMC4912640

[ref-61] Uliano-Silva M Ferreira JGRN Krasheninnikova K : MitoHiFi: a python pipeline for mitochondrial genome assembly from PacBio high fidelity reads. *BMC Bioinformatics.* 2023;24(1): 288. 10.1186/s12859-023-05385-y 37464285 PMC10354987

[ref-62] Vasimuddin M Misra S Li H : Efficient architecture-aware acceleration of BWA-MEM for multicore systems.In: *2019 IEEE International Parallel and Distributed Processing Symposium (IPDPS).*IEEE,2019;314–324. 10.1109/IPDPS.2019.00041

[ref-63] Weisz JB Lindquist N Martens CS : Do associated microbial abundances impact marine demosponge pumping rates and tissue densities? *Oecologia.* 2008;155(2):367–376. 10.1007/s00442-007-0910-0 18030495

[ref-64] Wu YW Tang YH Tringe SG : MaxBin: an automated binning method to recover individual genomes from metagenomes using an expectation-maximization algorithm. *Microbiome.* 2014;2(1): 26. 10.1186/2049-2618-2-26 25136443 PMC4129434

[ref-65] Zhou C McCarthy SA Durbin R : YaHS: Yet another Hi-C Scaffolding tool. *Bioinformatics.* 2023;39(1): btac808. 10.1093/bioinformatics/btac808 36525368 PMC9848053

