# Supplementary material for: The chromosomal genome sequence of the sponge Phakellia ventilabrum (Linnaeus, 1767) and its associated microbial metagenome sequences
Source: Wellcome Open Res. 2026 Jan 6;11:15. [Version 1] doi: 10.12688/wellcomeopenres.25431.1 (PMC12859430; doi:10.12688/wellcomeopenres.25431.1)
Supplement: Peer review history

# Reviewer report: Reviewer response for version 1

**DOI:** 10.21956/wellcomeopenres.28017.r144180

**Published:** 2026-01-30

**Authors:** Arzu Karahan

**Affiliations:** 1 Middle East Technical University, Erdemli-Mersin, Turkey

The research is well designed, thoroughly analyzed, and clearly presented. I have no criticisms regarding the analyses, writing, or data quality. However, it would have been beneficial to analyze eukaryotic metagenomes from species sharing the same environment (or living on or in it), such as Chordata, Cnidaria, and Echinodermata (Figure 3), and give at least the family- or genus-level taxa name.

 Metagenome raw data should also be released.

Are sufficient details of methods and materials provided to allow replication by others?

Yes

Is the rationale for creating the dataset(s) clearly described?

Yes

Are the datasets clearly presented in a useable and accessible format?

Partly

Are the protocols appropriate and is the work technically sound?

Yes

Reviewer Expertise:

metagenomics, transcriptomics, regeneration, aging, metabolomics, metabarcoding

I confirm that I have read this submission and believe that I have an appropriate level of expertise to confirm that it is of an acceptable scientific standard.

# Reviewer report: Reviewer response for version 1

**DOI:** 10.21956/wellcomeopenres.28017.r144173

**Published:** 2026-01-16

**Authors:** Poppy Hesketh-Best

**Affiliations:** 1 IGTP Institut Germans Trias i Pujol, Barcelona, Spain

This data note by Taboada and colleagues presents HiFi sequencing data for the marine sponge *Phakellia ventilabrum*. The authors generated a high-quality assembled and annotated sponge genome and further reconstructed bacterial metagenome-assembled genomes (MAGs) from the associated microbiome.

**Is the rationale for creating the dataset(s) clearly described?**

*P. ventilabrum* is a vulnerable marine ecosystem indicator species and is appropriately presented by the authors as a species of interest, particularly with respect to its potentially compromised resilience under climate change scenarios. In addition, its potential ability to accumulate environmental DNA (eDNA) broadens interest in research on genetic interconnectivity within this species and among surrounding taxa through eDNA-based studies. I am satisfied that the authors have presented a strong rationale for this dataset.

**Are the protocols appropriate and is the work technically sound?**

 I cannot comment on the methodology used for the sponge genome assembly due to a lack of relevant expertise. However, the metagenome-assembled genome (MAG) protocol follows established practices, quality assignment for the MAGS, employing commonly used workflows and software that are widely adopted by the broader field.

 It would be good for the authors to confirm their MAG QC using CheckM2 and the updated database, since the one used is 10 years old now. CheckM2 is more suited to MAGs with fewer representative species, which is likely the case with this deep-sea sponge species.

**Are sufficient details of methods and materials provided to allow replication by others?**

 The methods are well described and rely on open-source software, software containers, and provided detailed version controls. Several analyses are implemented using established workflows managed with Nextflow, enhancing transparency and reproducibility.

**Are the datasets clearly presented in a useable and accessible format?**

 Overall, the data are available in public repositories and were accessible as of 2026-01-15. However, none of the 33 metagenome-assembled genomes (MAGs) have been made publicly available. It is unusual that the sponge genome and its annotation are accessible, while the bacterial MAGs are not.

Are sufficient details of methods and materials provided to allow replication by others?

Yes

Is the rationale for creating the dataset(s) clearly described?

Yes

Are the datasets clearly presented in a useable and accessible format?

Partly

Are the protocols appropriate and is the work technically sound?

Yes

Reviewer Expertise:

Microbial genomics and bioinformatic

I confirm that I have read this submission and believe that I have an appropriate level of expertise to confirm that it is of an acceptable scientific standard.
